# Electron Concentration Limit in Ge Doped by Ion Implantation and Flash Lamp Annealing

**DOI:** 10.3390/ma13061408

**Published:** 2020-03-20

**Authors:** Slawomir Prucnal, Jerzy Żuk, René Hübner, Juanmei Duan, Mao Wang, Krzysztof Pyszniak, Andrzej Drozdziel, Marcin Turek, Shengqiang Zhou

**Affiliations:** 1 Institute of Ion Beam Physics and Materials Research, Helmholtz-Zentrum Dresden-Rossendorf, P.O. Box 510119, 01314 Dresden, Germany; r.huebner@hzdr.de (R.H.); juanmei.duan@hzdr.de (J.D.); m.wang@hzdr.de (M.W.); s.zhou@hzdr.de (S.Z.); 2 Institute of Physics, Maria Curie-Sklodowska University, Pl. M. Curie-Sklodowskiej 1, 20-035 Lublin, Poland; jotzet@hektor.umcs.lublin.pl (J.Ż.); krzysztof.pyszniak@poczta.umcs.lublin.pl (K.P.); adrozdzowa@o2.pl (A.D.); mturek@kft.umcs.lublin.pl (M.T.)

**Keywords:** Ge, ion implantation, flash lamp annealing, n-type doping, Raman spectroscopy

## Abstract

Controlled doping with an effective carrier concentration higher than 10^20^ cm^−3^ is a key challenge for the full integration of Ge into silicon-based technology. Such a highly doped layer of both p- and n type is needed to provide ohmic contacts with low specific resistance. We have studied the effect of ion implantation parameters i.e., ion energy, fluence, ion type, and protective layer on the effective concentration of electrons. We have shown that the maximum electron concentration increases as the thickness of the doping layer decreases. The degradation of the implanted Ge surface can be minimized by performing ion implantation at temperatures that are below −100 °C with ion flux less than 60 nAcm^−2^ and maximum ion energy less than 120 keV. The implanted layers are flash-lamp annealed for 20 ms in order to inhibit the diffusion of the implanted ions during the recrystallization process.

## 1. Introduction

It is postulated that the performance of the device in the next generation of nanoelectronics will be based on the implementation of these two tasks: (i) integration with semiconductors in CMOS technology characterized by carrier mobility that is much higher than in Si and (ii) further miniaturization [1]. Germanium is the most promising material for this purpose. The mobility of electrons and holes in Ge is two and four times higher than in Si, respectively. At the same time, Ge has very similar chemical and physical properties to Si, which significantly simplifies the integration process. Generally speaking, the use of germanium as a basic semiconductor in nanoelectronics would enable the production of faster devices maintaining the current technology [2,3]. However, the full integration of Ge into CMOS technology requires channel doping for both n- and p-type materials above 5 × 10^19^ cm^−3^ and contact doping above 10^20^ cm^−3^. It has been shown that p-type doping in Ge is less problematic, while n-type doping remains challenging. Al or Ga that are implanted into Ge and subjected to rapid thermal annealing (RTA) for tens of seconds or flash lamp annealing (FLA) for milliseconds act as acceptors at hole concentrations above 10^21^ cm^−3^ [4,5,6]. Such highly doped Ge exhibits superconductivity at critical temperatures below 1 K and it can provide excellent low resistive ohmic contacts. In case of n-type doping, P ions are the most attractive due to the highest solubility limit among other group V elements and relatively low activation energy [6,7,8,9]. However, conventional doping using P implantation that is followed by RTA or furnace annealing is limited to the concentration of about 5 × 10^19^ cm^−3^. Molecular beam epitaxy (MBE) enables in-situ doping within the range of 6 × 10^19^ cm^−3^ for the P ion doping layer, approximately 8 × 10^19^ cm^−3^ for As-doped Ge and slightly above 10^20^ cm^−3^ for Sb-doped Ge [9]. In the case of As and Sb, the level of doping is several times higher than the equilibrium solid solubility of As and Sb in Ge, which makes this material metastable. Milazzo et al. demonstrated that the post-grown thermal treatment of As and Sb at temperatures that are higher than 350 °C, e.g., during contact formation, reduces the effective concentration of electrons to the level of 2–3 × 10^19^ cm^−3^ [10]. P-doped Ge is much more thermally stable. The equilibrium solubility of P in Ge at 800 °C is about 2 × 10^20^ cm^−3^ and might be even higher at a temperature close to the melting point of Ge. Unfortunately, at high temperature, a strong diffusion of dopants takes place, which widens the depth distribution of donors. According to the literature, the maximum concentration of electrons in Ge increases with decreasing thickness of the doping layer, regardless of doping techniques [11]. For example, using the MBE method and delta doping, if the thickness of the doping layer is in the range of a few nm, the effective concentration of the carriers can be as high as 2 × 10^20^ cm^−3^ [12]. P-implanted Ge shows the highest carrier concentration of approximately 6 × 10^20^ cm^−3^ for a layer thickness of around 40 nm [11]. Kujala et al. examined the deactivation of donors in strongly doped Ge using positron annihilation spectroscopy [13]. They demonstrated that the deactivation of donors in Ge is mainly caused by mono- and divacancies. In highly doped Ge, a single open volume defect can be decorated by four donors, which makes them electrically inactive. According to theoretical calculations, such defect centers, i.e., vacancies that are associated with donors (V_n≥1_D_1≤m≤4_) are not thermally stable [14,15,16]. Chroneos and Bracht demonstrated that, after annealing at the temperature above 700 °C, most of the V_n_D_m_ clusters dissolve [16]. Unfortunately, strong diffusion of donors is activated during conventional high temperature annealing, which widens their distribution. Moreover, vacancies that are released from the clusters are again trapped by the donors, which reduces the effective concentration of the carriers to the equilibrium level, which is approximately 2–3 × 10^19^ cm^−3^ for Ge.

The second fabrication challenge for highly doped Ge and GeSn alloys using ion beam implantation is related to surface degradation and the formation of large open volume defects within the implanted layer. Such defects are thermally stable and they cannot be removed from the layer during solid phase epitaxy. During the implantation process, Ge atoms diffuse through vacancies towards the surface [16]. It has been shown that high-fluence ion implantation causes surface roughness that is proportional to the mass, energy, and fluence of the ions. The surface roughening can be partially suppressed by low temperature ion implantation (<−100 °C) and a protective layer. Tran et al. demonstrated that the 50 nm thick SiO_2_ layer is sufficient for maintaining a flat surface of Ge during the implantation of Sn to fluence of 4 × 10^16^ cm^−2^ with energy of 100 keV, which corresponds to the concentration of Sn even up to 15% [17]. Subsequently, high quality GeSn alloys were made while using pulse laser annealing. A similar surface quality can be obtained by using a protective layer of Si or SiN for Sn fluences up to 1 × 10^16^ cm^−2^ [17]. At higher fluences, voids were observed within the implanted layer. Apart from the capping layer, the key parameter preventing the surface degradation of Ge is the ions flux, which must be sufficiently low to prevent the surface from heating up from energetic ions during the implantation. 

In this work, the influence of implantation and annealing parameters on the degradation of Ge surface and the activation efficiency of donors in Ge is examined. It is shown that for P-ion fluences up to 1 × 10^15^ cm^−2^ with an ion flux up to 60 nAcm^−2^ and an ion energy up to 120 keV, low-temperature ion implantation (<−100 °C) is sufficient for preventing surface degradation. For higher fluxes and/or heavier ions, such as As, Sb, or Sn, the Ge surface must be protected with a capping layer and the surface temperature must be kept below −100 °C. The effective electron concentration in P-implanted Ge is strongly dependent on the P concentration and the thickness of the implanted layer. The highest electron concentration is achieved with a doped layer of 40 nm thick. Whereas, the electron concentration is in the range of 6 × 10^20^ cm^−3^ and it drops down to 8–9 × 10^19^ cm^−3^ for doped layers that were thicker than 300 nm. Moreover, we show that recoil implantation of the atoms from the capping layer to Ge should be considered. A co-implantation of Si and O takes place in the case of the SiO_2_ capping layer. During the post-implantation annealing, O is bonded to Ge and forms GeO_x_ nanoclusters, which might cause the deterioration of electrical and optical properties of the produced layer.

## 2. Materials and Methods

Intrinsic Ge wafers were implanted with P, As, Sb, Ga, and Sn ions. The implantation energy was chosen to obtain the doped layer up to 300 nm thick. The ion fluence was varied between 1 × 10^14^ cm^−2^ to 4 × 10^16^ cm^−2^, which corresponds to the maximum concentration of dopants in the range from 1 × 10^18^ cm^−3^ to 4 × 10^21^ cm^−3^. The ion current flux was in the range from 10 to 500 nAcm^−2^. In order to protect the surface from degradation and contamination during sample processing 30 nm thick SiO_2_ or Si layers were used. The ion range and dopant concentration were calculated while using the SRIM code [18] and experimentally confirmed by random and channeling Rutherford backscattering spectrometry (RBS/R and RBS/C). In particular, RBS was carried out using the 1.7 MeV He^+^ beam of the Rossendorf van de Graaff accelerator. The dopant profiles (and the thickness of the doped layer) were then calculated with the help of the RUMP program [19]. RBS was only used to determine the distribution of Sn and Sb in Ge due to the resolution limits for dopants lighter than the matrix. In the case of the P, As, or Ga implanted layer, RBS/R and RBS/C were used to assess the thickness of the implanted layer from as-implanted samples. In such a case, the RBS/C spectra obtained from implanted region shows the same yield like the RBS/R signal. Next using RUMP software the thickness of the implanted layer can be calculated. RBS/R and RBS/C were also used to estimate the recrystallization efficiency after annealing. After the ion implantation, the samples were subjected to thermal treatment while using a flash annealing system operating in millisecond regime [20] or pulsed laser melting using a pulse length of 28 ns of an excimer laser (308 nm wavelength). Next, the SiO_2_ capping layer was removed using diluted hydrofluoric acid (HF). The carrier concentration in the implanted and annealed samples was estimated from temperature-dependent Hall effect measurements in the van der Pauw configuration. Cross-sectional bright-field and high-resolution transmission electron microscopy (TEM,) investigations were performed on a Titan 80–300 (FEI, Eindhoven, Netherlands) microscope operating at an accelerating voltage of 300 kV to investigate the microstructural properties of the implanted Ge layer. High-angle annular dark-field scanning transmission electron microscopy (HAADF-STEM,) imaging and spectrum imaging analysis based on energy-dispersive X-ray spectroscopy (EDXS) were performed at 200 kV with a Talos F200X microscope (FEI, Brno, Czech Republic) equipped with a Super-X EDXS detector system (FEI). Prior to TEM analysis, the specimen that was mounted in a high-visibility low-background holder was placed for 10 s into a Model 1020 Plasma Cleaner (Fischione, Export, PA, USA) to remove possible organic contaminations. The surface topography of the implanted Ge was investigated while using top-down scanning electron microscope (SEM), which was performed using a S-4800 microscope (Hitachi, Tokyo, Japan) that was operating at an accelerating voltage of 10 keV. The phonon spectra and recrystallization efficiency of the implanted and annealed layers were measured by room-temperature micro-Raman spectroscopy, where the sample was excited with a green laser (λ = 532 nm) and the signal was recorded with liquid nitrogen cooled silicon CCD camera (Horiba, Bensheim, Germany). 

## 3. Results

### 3.1. Microstructural Properties

**Figure 1:**
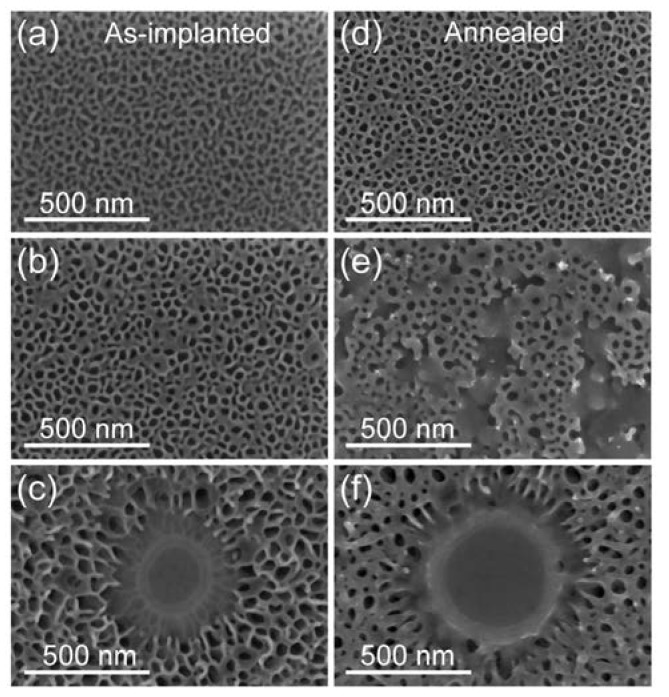
Shows top-down scanning electron microscope (SEM) images of as-implanted (**a**–**c**) and annealed (**d**–**f**) Ge wafers. The samples were implanted using Sn^+^ ions with three different concentrations: 0.5% (**a**,**d**), 1% (**b**,**e**), 1.5% (**c**,**f**). The annealing was made using flash lamp annealing (FLA) for 3 ms at 41 Jcm^−2^.

Figure 1 shows top-down SEM images that were obtained from unprotected Ge samples implanted with Sn^+^ ions at low temperature before (a–c) and after FLA (d–f) for 3 ms at an energy density of 41 Jcm^−2^. The Sn concentrations/fluences are as follows: 0.5 at.%/1.4 × 10^15^ cm^−2^ in (a) and (d), 1 at.%/2.8 × 10^15^ cm^−2^ in (b) and (e) and 1.5 at.%/4.2 × 10^15^ cm^−2^ in (c) and (f). During the implantation process, the sample temperature measured at the sample holder was kept below −120 °C and the ion flux was about 350 nAcm^−2^. The Sn ions were implanted with an ion energy of 150 keV. Here, Sn is used as an example ion, since the same surface degradation was obtained for Sb and As doping (not shown here). In the case of P-implantation, the surface degradation of Ge is a bit smaller and it appears for slightly higher fluencies, but the physical phenomena are the same.

**Figure 2:**
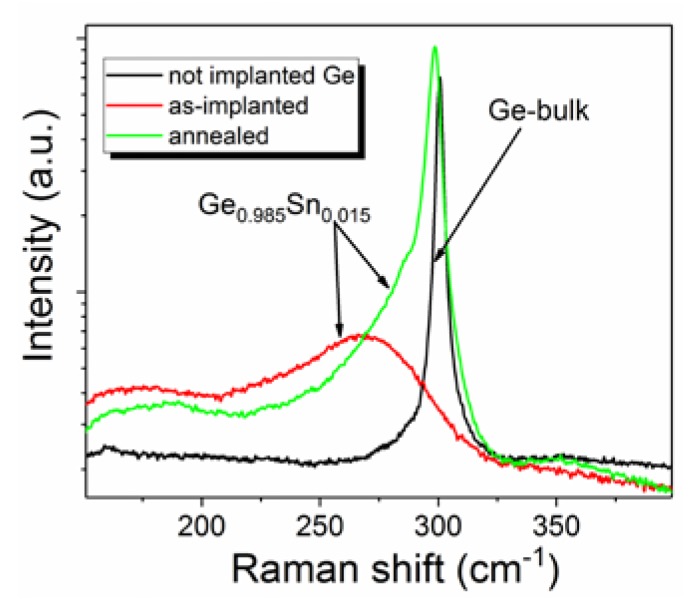
Micro-Raman spectra obtained from as-implanted and from annealed Ge wafers that were implanted with Sn. The Sn concentration in the layer is 1.5%. The spectrum from intrinsic bulk Ge is shown for the reference.

Figure 1a–c show the increases of the surface roughness with the ion fluence. For the highest fluence, we have found not only three-dimensional roughening, but also the formation of circular islands is shown. Some of such islands were already observed for the middle fluence, but the diameter and the density of the islands are much smaller than for the highest Sn fluence. Most probably, the islands are formed at places where few of the nanostructures connect together and Ge is transported to the surface of the nanostructures due to anomalous plastic deformation in the amorphous phase. A similar effect was observed in ion-irradiated Si nanowires (NWs) at room temperature [21]. The plastic deformation of Si NWs is suppressed by increasing the sample temperature above 350 °C, which was the threshold temperature for avoiding NW amorphization during the implantation process. Additionally, ion irradiation of Ge at an elevated temperature can be used to control the nanostructure formation. It was shown that high-fluence ion irradiation of Ge at an elevated temperature fully suppresses the amorphization and causes reverse epitaxy [22]. Figure 1d–f show the surface of the implanted Ge after FLA for 3 ms at the energy density of 41 Jcm^−2^. According to Raman spectroscopy, the annealed samples are crystalline and Sn is incorporated into the Ge lattice (see Figure 2). The main phonon mode in GeSn is located at 298.6 cm^−1^. Using the formula Δω = −ω_Ge_ = c × x_Sn_ where c is the constant −82.8 (taken from Ref. 23), x_Sn_ is Sn concentration, ω_GeSn_ and ω_Ge_ are the transverse optical (TO) phonon mode position in GeSn and bulk-Ge, respectively, and, while assuming fully relaxed layer, we can calculate the Sn concentration. Based on the Raman spectrum, the Sn concentration in presented film is in the range of 2.4%, which is much higher than expected from implantation parameters. In this simple calculation, the strain is not taken into account. Since we know the Sn concertation from independent methods (in this sample Sn concentration is 1.5%), using Raman spectroscopy it is possible to estimate the strain in the layer while using formula Δω = a × x_Sn_ + b × ε, where ε is the strain, a = −83.11 and b = −374.53 (after Ref. [23]). According to the calculation, the honeycomb structure that is presented in Figure 1f is tensile strained, with ε = 0.86%. The recrystallization of the implanted layer is due to the solid phase epitaxy. However, the honeycomb structure is preserved, which significantly limits the potential application of such a material. Therefore, the prevention of the surface degradation during ion implantation is crucial in device performance. 

**Figure 3:**
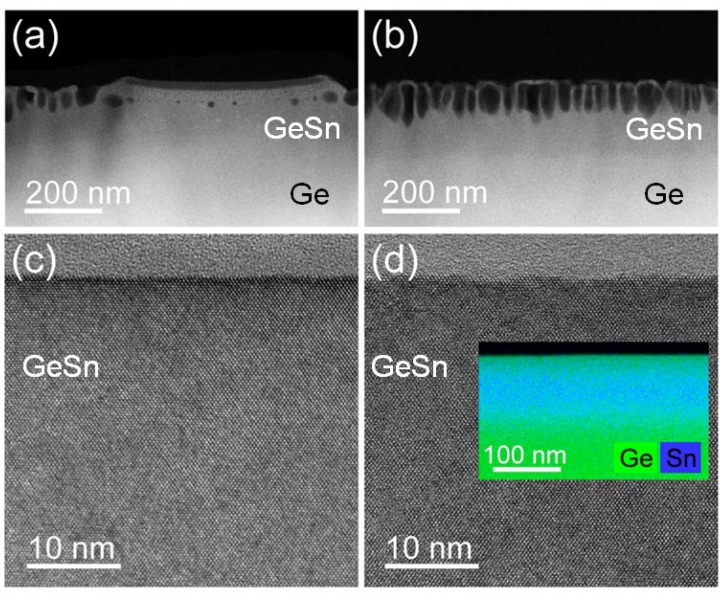
(**a**,**b**) show cross-sectional scanning transmission electron microscopy (STEM) images obtained from the samples presented in Figure 1 (**b**) (fluence: 2.8 × 10^15^ cm^−2^) and (**c**) (fluence: 4.2 × 10^15^ cm^−2^) for the island region and next to it, respectively. (**c**) shows a cross-sectional high-resolution TEM image obtained from Sn-implanted Ge (fluence: 2.8 × 10^15^ cm^−2^) without capping layer after PLM and (**d**) shows a high-resolution TEM image of a Sn-implanted sample (fluence: 1.2 × 10^16^ cm^−2^, energy: 200 keV) with SiO_2_ capping layer and with low ion flux after FLA. The inset in (**d**) shows the Sn distribution (blue color) in Ge (green color) based on energy-dispersive X-ray spectroscopy (EDXS) analysis in scanning TEM mode.

Figure 3a,b show cross-sectional HAADF-STEM images of the Sn-implanted Ge representing the regions of an island and next to it, respectively. It is clear that, within the island, the Ge forms a continuous layer, but voids are well visible below the surface. In general, the height of the honeycomb structure increases with increasing the ion fluence at the same ion energy. In our case, for the smallest Sn fluence of 1.4 × 10^15^ cm^−2^, the height of the honeycomb structure varies between 30 and 80 nm. It increases slightly above 100 nm for the middle fluence of 2.8 × 10^15^ cm^−2^ and it is above 120 nm for the Sn fluence of 4.2 × 10^15^ cm^−2^. In general, the implanted layers can be recrystallized while using either solid phase epitaxy or liquid phase epitaxy. Gao et al. have shown that, while using pulsed laser melting, even such a rough surface like that presented in Figure 1b can be removed [24]. Figure 3c presents a cross-sectional high-resolution TEM image that was obtained from Sn-implanted Ge with a fluence of 2.8 × 10^15^ cm^−2^ without capping layer after pulsed laser melting. During melting within nanoseconds, the Ge surface becomes flat, and the implanted layer recrystallizes via liquid phase epitaxy. Figure 3d shows a high-resolution TEM image obtained from Ge that was implanted with Sn using a 30 nm thick SiO_2_ layer pointing to solid phase epitaxy during 3 ms FLA. The SiO_2_ capping layer was removed after FLA while using wet etching in diluted HF. The Sn fluence was 1.2 × 10^16^ cm^−2^ and the ion energy was 200 keV. Hence, the Sn fluence is almost ten times higher than that presented in Figure 1a,d, but the Ge surface was perfectly protected from sputtering and roughening while using low-temperature ion implantation and a SiO_2_ capping layer. Moreover, the inset in Figure 3d shows that the diffusion of Sn can be fully suppressed using ms-range solid phase epitaxy. We have observed the same effect for other elements implanted into Ge, like Al, Ga, or P [6,7,25].

**Figure 4:**
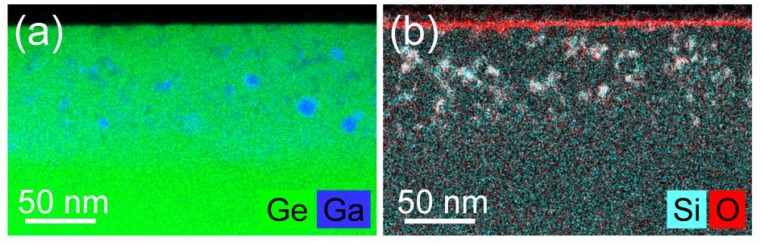
Depth distribution of Ga in Ge (**a**) and Si and O (**b**) in Ge heavily doped with Ga after ion implantation and flash lamp annealing. The elemental depth distributions are based on EDXS analysis in scanning TEM mode.

In the literature, the function of the capping layer is mainly discussed from the point of view of surface degradation of the implanted material [17]. However, the cross-contamination of the implanted materials with capping layer atoms is often very critical for the device performance. In the case of Ge, the cross-contamination with a small amount of Si can be neglected, but even a small concentration of O in Ge can significantly deteriorate the transport properties. While using SiO_2_ as the capping layer for high-fluence ion implantation, we have to consider the recoil implantation of Si and O into Ge. This aspect has been neglected by most of the researchers, and, to the best of our knowledge, the O distribution within the implanted Ge layer has never been reported so far. Figure 4 shows the distribution of Ga (a) and Si and O (b) in Ge implanted with Ga to the fluence of 4 × 10^16^ cm^−2^ through a 30 nm thick SiO_2_ layer. The sample was annealed for 20 ms while using flash lamp annealing with an energy density of 120 Jcm^−2^ and the capping layer was removed after annealing using buffered hydrofluoric acid. The thickness of the doped layer is around 100 nm with a Ga concentration approximately 4 × 10^21^ cm^−3^, which is eight times more than the equilibrium solid solubility of Ga in Ge. 

For such high doping level, even ms-range annealing is not able to fully suppress the Ga-cluster formation during the recrystallization process. A part of Ga is agglomerated within metallic Ga clusters with an average diameter of about 10 to 20 nm. Moreover, many of the Ga-containing clusters have irregular shapes. Those clusters are composed of Ga and Ge, with the Ga content being much higher than the average doping in the surrounding. Interestingly, the element distributions of Si and O match quite well, which points to a possible formation of Si-Ge-O clusters (see Figure 4b). A small amount of Si added into Ge can slightly reduce the carrier mobility, but oxide clusters do strongly affect the carrier transport. Amorphous Si, which can be removed selectively from Ge using wet-chemical etching e.g., using tetramethylammonium hydroxide (TMAH), would just be an alternative for the SiO_2_ capping layer. Therefore, the capping layer must be properly selected when the experiment is designed. 

### 3.2. Optical and Electrical Properties

**Figure 5:**
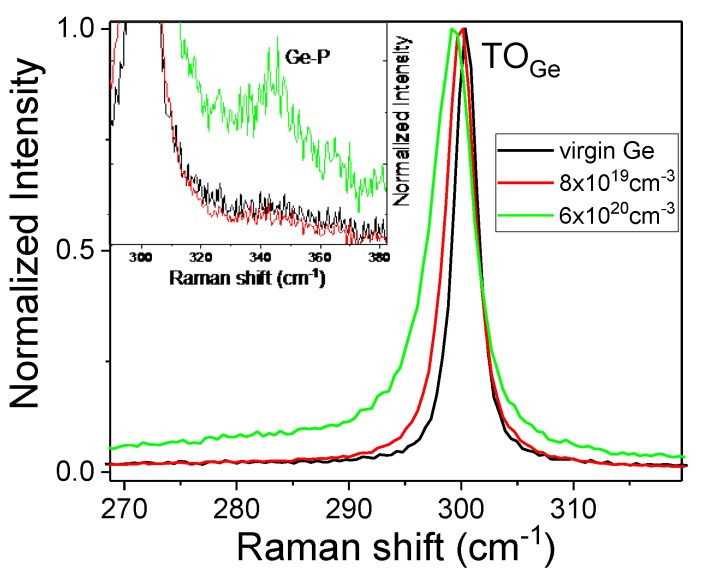
Normalized Raman spectra obtained from intrinsic and P-implanted Ge after FLA for 20 ms. The admixture layer thickness is either 40 or 300 nm and the maximum carrier concentration is 6 × 10^20^ cm^−3^ and 8 × 10^19^ cm^−3^, respectively. The insert shows the enlarged area, where the Ge-P vibrating mode is expected.

Figure 5 shows the micro-Raman spectra that were obtained from intrinsic and P-implanted Ge after flash lamp annealing for 20 ms at an energy density of 120 Jcm^−2^. The thickness of the doped layer is either 300 nm or 40 nm and the maximum carrier concentrations are about 8 × 10^19^ cm^−3^ and 6 × 10^20^ cm^−3^, respectively. The P concentration has been optimized to obtain maximum carrier concentrations for each layer thickness. For the thick layer, the P concentration is 1 × 10^20^ cm^−3^ and for the thin layer 1 × 10^21^ cm^−3^.

The transverse optical (TO) phonon mode in intrinsic Ge is observed at 300.5 cm^−1^ [23], which means that the test sample is fully relaxed. After P-doping, the TO phonon mode moves significantly towards the lower wavenumber and the full width at half maximum (FWHM) increases. What is more, the peaks in the spectrum of implanted layers are asymmetrical. The peak widening and red shift of the TO phonon mode results from the interaction of phonons with carriers, the so-called Fano effect [26,27]. The Fano effect is easily observable in heavily doped p- and n-type semiconductors. The inset in Figure 4 shows the local vibration phonon mode observed between P and Ge atoms. The Ge-P phonon mode is only visible in very heavily doped Ge. The existence of this vibration mode confirms that most of the P atoms are located at the substitutional position. 

**Figure 6:**
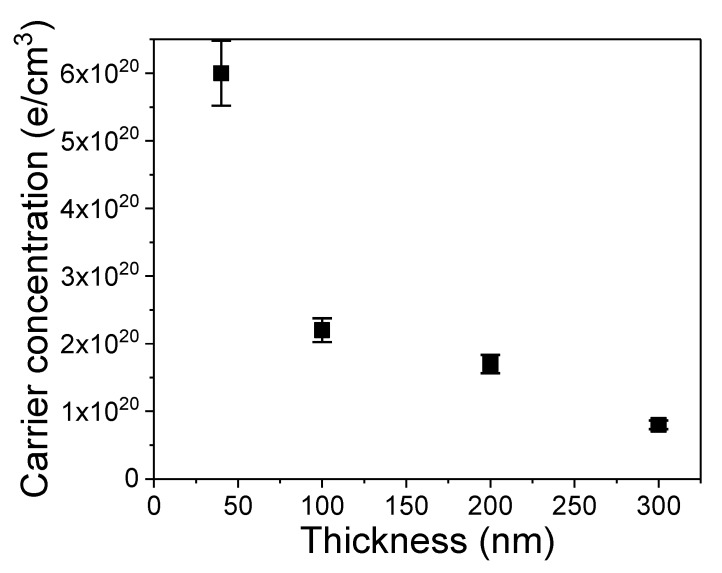
The maximum carrier concentration estimated from Hall effect measurements as a function of the thickness of the doped layer in P-implanted Ge after FLA for 20 ms with an energy density of 120 Jcm^−2^.

Figure 6 shows the maximum carrier concentration that was obtained from Ge implanted with P ions followed by FLA for 20 ms. The samples were implanted at a temperature lower than −120 °C through the SiO_2_ layer. The values of energy and fluences of the implanted P^+^ ions were optimized for each sample. For thicker layers the P concentration varied between 5 × 10^19^ cm^−3^ and 5 × 10^20^ cm^−3^ and for the 40 nm thick Ge layer the maximum P concentration was 1.5 × 10^21^ cm^−3^. In the case of thin films, the carrier concentration continuously increases with the P concentration up to the doping level of approximately 1 × 10^21^ cm^−3^. For a P concentration higher than 1 × 10^21^ cm^−3^, the effective electron concentration significantly decreases, which is most probably due to the formation of P-dimers and P-clusters. P-dimers in Ge are deep donors and P-clusters are electrically not active. 

In the case of thicker layers, we have observed the s−aturation of the maximum carrier concentration for a P concentration of about 1 × 10^20^ cm^−3^ for 300 nm thick Ge and of about 4 × 10^20^ cm^−3^ for 100 nm thick layer. In each case, the increase of the P concentration decreases the maximum electron concentration. Vohra et al. have shown that, in n-type Ge, the Ge-vacancies are mainly responsible for the deactivation of donors [28]. They can form donor-vacancy clusters and, for heavily doped systems, one vacancy can deactivate up to four donors. We assume that during very short annealing at high temperature we can dissolve some of such defect centers and, in a very thin layer (thickness in the range of 40 nm or less), vacancies can diffuse out of the doped layer releasing electrically active donors. However, in thicker layers, they can still find new dopants and form new vacancy-donor complexes. One solution would be to increase the annealing time that vacancies would have more time for out-diffusion. Unfortunately, the donor diffusion in Ge is vacancy-mediated and known as a ring diffusion mechanism [16]. The prolonged diffusion would remove more vacancies from the doped layer, but simultaneously donors will also diffuse and will be trapped by new vacancies. Hence, the maximum carrier concentration in n-type Ge strongly depends on the thickness of the doped layer. Fortunately, nowadays, nanoelectronics require an ultra-doped layer in the thickness range of a few to tens of nanometers. This makes ion implantation followed by ms-range FLA one of the most promising doping technique for high-performance nanoelectronics.

## 4. Conclusions

We have demonstrated that the surface degradation of Ge during high-fluence ion implantation can be significantly suppressed by using a capping layer and performing the ion implantation at cryogenic temperatures. The material of the protective layer must be carefully selected due to potential cross-contamination. We have shown that the maximum carrier concentration in n-type Ge depends not only on the implantation and annealing parameters, but also on the thickness of the doping layer. The maximum electron concentration has been found to be in the range of 6 × 10^20^ cm^−3^ for 40 nm thick implanted Ge layers.
